# Health information needs and feedback of users in the online TCM community

**DOI:** 10.1371/journal.pone.0301536

**Published:** 2024-03-29

**Authors:** Lei Nie, Jiayi Xu, Ruojia Wang

**Affiliations:** 1 Country and Area Studies Academy, Beijing Foreign Studies University, Beijing, China; 2 International Business School, Beijing Foreign Studies University, Beijing, China; 3 School of Management, Beijing University of Chinese Medicine, Beijing, China; University of Tartu, ESTONIA

## Abstract

To improve the information service quality of the online Traditional Chinese Medicine (TCM) community, this study investigated users’ information needs, feedback and the relationship between them. Using qualitative content analysis, the basic characteristics of users’ needs were obtained. Logistic regression was used to explore the impact of different need characteristics of feedback. The main findings are as follows: 1) Disease consultation, health preservation, professional discussion, knowledge sharing and experience description are the major 5 types of information needs in the online TCM community; 2) Some users provided TCM-related information, such as the tongue image and the TCM four diagnosis; 3) A total of 78.8% of the posts received effective feedback, and the main types of feedback were answering, discussing, inquiring and emotional supporting; 4) Providing enough information can significantly and positively affect whether needs receive effective feedback, suggesting that users can present information about their condition in as many different formats as possible when articulating their needs.

## Introduction

With the improvement of people’s living standards, public health literacy is on the rise. The 50th "Statistical Report on Internet Development in China" shows that as of September 2022, the number of internet users in China has reached 1.051 billion, of which the number of online medical users has reached 298 million [1]. As the earliest service in internet medical care, the online health community (OHC) refers to an open network platform that provides health consumers with information exchange, experience sharing, question-and-answer consultation and social support on health-related issues [2]. Obtaining health information through OHCs has become an important way for internet users to obtain health education and improve health information literacy [3,4]. For the general public and patients, especially those with chronic diseases, the powerful communication and interaction function of OHCs has an important positive impact on their self-managed health and daily disease control [5,6].

Traditional medicine is broadly used across Asian societies [7]. Traditional Chinese Medicine (TCM), the origins of which can be traced back thousands of years, has a culturally significant role as a traditional medicine among the Chinese population [8]. It is not only applied for daily health maintenance but also used in alternative treatments for cancers, COVID-19, and many other medical conditions [9,10]. At present, TCM is widely used in China, among Chinese immigrants and is even prevalent in other countries [11,12].

Health information needs are the starting point and motivation for a series of health information behaviors by online health community users. Different types of users (e.g., elderly individuals, pregnant women and adolescents) use online health communities to consult, communicate and interact with each other, resulting in a large amount of user-generated content that is an important source of data to understand users’ health information needs [13,14]. The behavior of different types of users in online health communities has its own characteristics. Previous studies show that there is a difference between TCM-related health information behaviors and Western medicine performance [15,16], but these studies are only reflected in the results, and there is no targeted research on TCM-related online health communities.

To further promote the construction of an online TCM community, this study selected online health community platforms for TCM and analyzed the posts and replies of TCM community users from three perspectives: information needs, feedback, and the influence of needs on feedback. The specific research questions are as follows.

RQ1. Perspective of information needs: What are the types of information needs of online TCM community users? How do users express and describe their information needs?
RQ2. Perspective of users’ feedback: How effective are the responses provided by responders in addressing the information needs of users within TCM online health communities? What are the types of responses offered by responders to address users’ information needs within the community?
RQ3. Perspective of influencing factors: What characteristics of the expression of information needs affect whether they can be effectively fed back?

## Literature review

Health information needs are an important research topic in the field of information behavior. Related research has always focused on a specific population because health information needs vary among people with different health statuses. For common chronic diseases, a survey of hypertensive patients showed that blood pressure monitoring, medication reminders, and hypertension education are the most important information needs among patients with hypertension at eight Chinese primary medical units [17]. Additionally, for diabetes patients, social support categories identified in users’ posts showed that they are most interested in achievement, congratulations, network support, seeking emotional support, seeking informational support and so on [18]. For critical diseases, a text mining study showed that worrying about cancer with subjective symptoms occurs most frequently among all breast cancer patient posts [19]. A qualitative study summarized information needs of individuals who have sexually transmitted infections in a web-based forums of the American Sexual Health Association, which found that psychosocial information need should be caught more attention [20]. A study focused on patients with albinism identified 8 main topics discussed in OHCs: daily sharing, family, interpersonal communication, social life and security, medical care, occupation and education, beauty, and self-care. Among these topics, daily sharing represented the largest proportion of the discussions [21]. In addition, the information needs of some other diseases, such as depression and COVID-19, have also been analyzed in detail [22–24].

With the improvement of living standards and the transformation of health concepts, consumers’ health information needs are no longer limited to specific diseases. Disease prevention and health care have received increasing attention in China. The tenets of TCM, in which the key concept of health preservation is preventing disease before its occurrence, are widely believed and applied in China, among Chinese immigrants and is even prevalent in other countries [25,26]. A study compared the distribution of topics between Western medicine posts and TCM posts and found that the proportion of TCM posts is higher for coping with aging and physical exercise, whereas the proportion of dietary nutrition posts is lower [16]. Another study revealed some differences between Western medicine posts and TCM posts in informational replies and emotional replies and found that compared to TCM posts, Western medicine posts received more informational replies but fewer emotional replies [15]. These results may indicate that users in different medical systems have different behaviors and information needs. However, compared with health information behavior, the research focus on TCM information behavior is relatively limited. In the Western medical system, TCM is usually classified as complementary and alternative medicine (CAM). Few studies have examined CAM information-seeking behaviors based on surveys or interviews [27–29], and even fewer have been based on data from online TCM communities.

More user interaction and feedback are proven to help further the development of OHCs or even improve the therapeutic remedies for some diseases. For instance, studies focusing on diabetes [30] have highlighted the significant role of user engagement in enhancing health outcomes. Additionally, a recent research explores the effects of interaction and intervention in social media on adolescents with drug use, underscoring the broader impact of user interaction in online platforms [31]. Therefore, some studies have explored the feedback received as well as health information needs. A previous study summarized the common replies in a social Q&A community to understand what types of support people are providing to and receiving from the community [32]. A content analysis study not only identified the types of questions that patients with diabetes and hepatitis are most concerned about but also determined the relationship between the characteristics of questions and the number of answers received. The results showed that the number of words per question and the value of the reward were negatively correlated with the number of answers [33]. Other studies explored the interaction mode between the consultant and the respondent in OHCs and found that the information seeker-perceived helpfulness does not depend on who answered the question but on how an information seeker posted the question [34]. A similar conclusion was reached in a study of patients with HPV, which found that both content length and vividness were positively related to the response behaviors of HPV vaccine-related answers [35].

Overall, the following gaps still exist in the study of health information needs. First, health information needs based on OHCs have been well studied in common diseases such as diabetes and cancer, but less is known about the information needs of users in online CAM communities. Second, previous studies have rarely investigated feedback on users’ health information needs. Most studies focus on how users express and describe their information needs, ignoring whether they receive effective replies or feedback on their information needs. Therefore, this study takes the online TCM community as an example and explores health information needs and feedback by analyzing posts and replies to provide a basis for improving online TCM health information services.

## Methods

**Fig 1:**
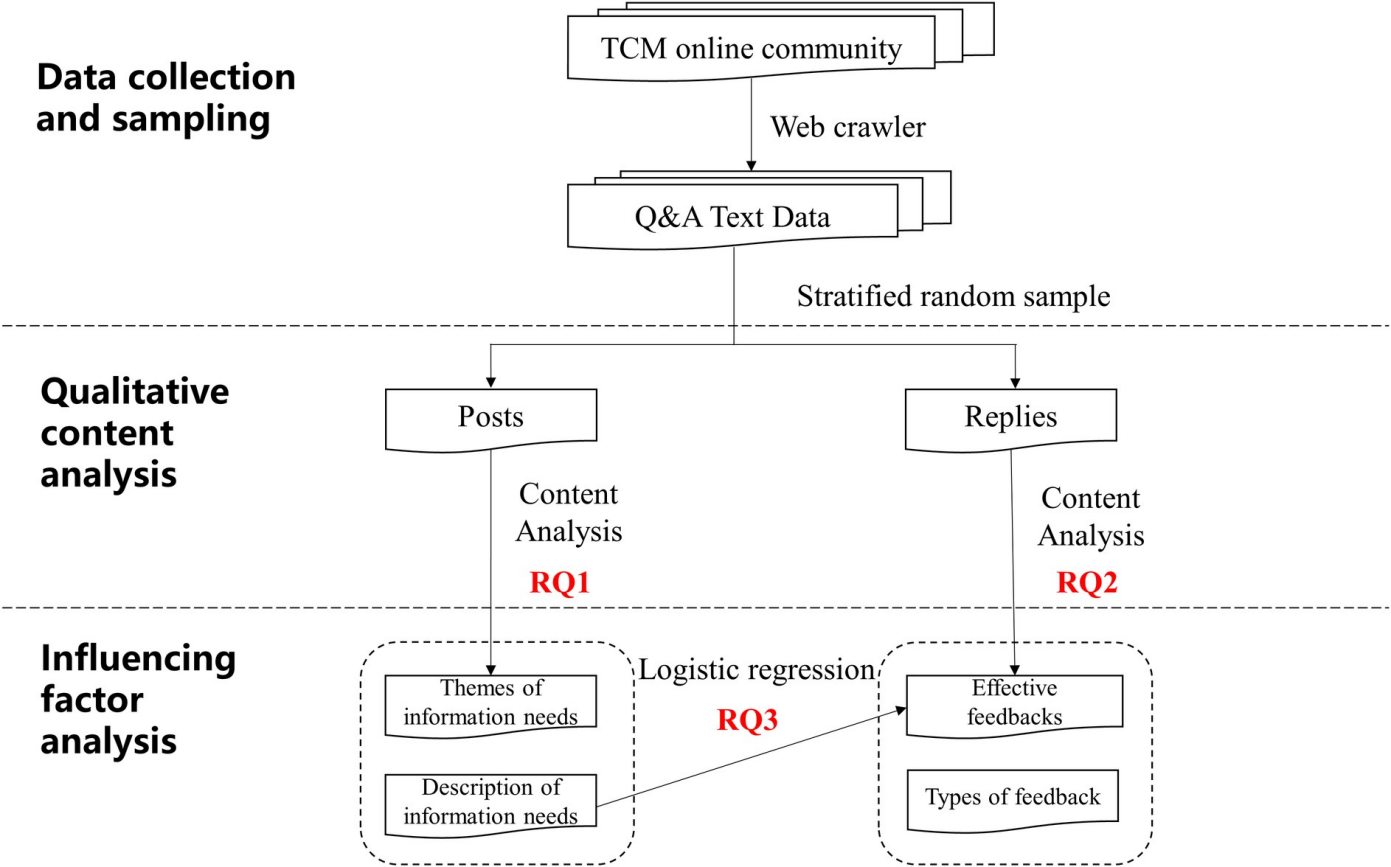
Research design and technical route.

The technical roadmap of this study is shown in Fig 1 and includes three parts: data collection and sampling, qualitative content analysis, and influencing factor analysis.

### Data collection and sampling

Posts and replies were carefully sampled from the TCM online community, ensuring that the sample was representative and diverse.

#### Qualitative content analysis

(1) To answer RQ1, the posts were coded by researchers based on their content and themes which provide insights into the main information needs within the online community. (2) For RQ2, the replies were similarly coded, focusing on the type of response and whether their replies effectively addressed the stated needs.

#### Influencing factor analysis

In addressing RQ3, regression analysis was employed to explore the influencing factors that may have influenced the quality of the interactions and the effectiveness of the response provided.

### Data collection and sampling

The Huaxia TCM Forum (http://www.hxzylt.com/) is a professional online communication forum for TCM in China that was created in 2008 and includes several modules, such as classical TCM and modern medical case studies, modern TCM clinical applications, inheritance, and comprehensive discussion. In this study, the disease consultation section of the comprehensive discussion module of the Chinese Medicine Forum was selected as the data source for the online TCM health community for the following reasons: (1) The forum was founded earlier, has tens of thousands of registered members, and has a great influence among Chinese people worldwide; (2) there are more posts in the comprehensive discussion module compared with other modules; and (3) the disease consultation section is an important place for professional‒patient communication, which helps identify the health information needs of patient-type users.

Huaxia TCM Forum is an openly accessible website. Moreover, this research only uses the information in the public text on the website and does not involve personal information. So the data collected from our web scraping activities do not involve any personal privacy concerns, making formal ethical approval unnecessary, and aligning with relevant requirements.

The Q&A text data of the disease consultation board were collected using Python, and a total of 11,075 posts and 115,513 replies were obtained. Due to the specialized nature of Traditional Chinese Medicine (TCM) and its unique language characteristics, quantitative feature extraction methods may not adequately capture the inherent in TCM discourse. Therefore, we adopted a qualitative approach to the analysis, recognizing the need for sampling due to the impracticality of analyzing the entire dataset comprehensively.

This study requires professionals in the field of TCM to manually annotate the text information in the forum, which is very labor- and time-consuming, and it is unlikely to be possible to annotate all of the posts and replies, so it is necessary to randomly select a sample from all of the messages. To ensure the validity and rigor of our sampling process, we implemented several measures. First, we developed a carefully designed stratified sampling method, taking into account various factors such as the number of replies and clicks. From this methodology, we extracted 1,000 posts along with their corresponding 9,951 replies to serve as the sample data for content analysis. As illustrated in Table 1, there is no significant difference between the two datasets in terms of the average number of clicks, replies, posting words, and reply words, which means that the sample data adequately represents the initial dataset. Subsequently, we used this sample for coding and conduct a saturation test to ensure a sufficient sample size. Further details regarding the coding and saturation test processes will be provided in subsequent sections.

**Table 1:** Basic statistics of the initial data and sample data.

Dataset	Number of clicks (Mean±Var)	Number of replies (Mean±Var)	Number of words posted (Mean±Var)	Number of words in reply (Mean±Var)
initial data	551.1±192.4	14.3±19.8	345.8±479.5	146.2±285.2
sample data	547.2±205.1	13.1±17.1	343.1±480.5	151.5±245.9

### Qualitative content analysis

This study uses qualitative content analysis to analyze users’ health information needs, which is based on inductive techniques to systematically encode and classify text content by summarizing it to identify the themes and patterns implied in the text [36].

Out of the 1,000 posts, 900 were utilized for coding, while the remaining 100 posts were reserved for the theoretical saturation test. All posts were open-coded from two perspectives: information needs and description of information. Due to the large volume of textual information, independent coding is not efficient, so 3 coders were involved in coding the data. To ensure the scientificity and consistency of the coding results, first, 3 coders coded the same 50 samples; there was much communication and discussion during the coding process, and if there was a dispute, the fourth person determined the final coding results. Then, 3 coders coded the other 50 posts back to back and tested the degree of coding consistency. The calculated kappa values are 0.81 and 0.79, both greater than 0.7, indicating strong coding consistency among them. After coding 900 posts, we developed a coding book summarizing the categories and themes (Table 2). Subsequently, we conducted a theoretical saturation test using the final 100 posts to ensure that no new concepts emerged.

**Table 2:** Part of the coding book of content analysis.

Content category	Coding	Explanation of codes
Demander	Patient	The post is initiated by patients or their relatives
	Professional	The post is initiated by doctors or other professionals
Information needs	Disease consultation	The post seeks consultation or information related to a specific disease
	Health preservation	The post seeks information or advice related to daily health routines or habits
	Experience description	The post shares personal experiences related to health or treatment
	Professional discussion	The post initiates a discussion among healthcare professionals
	Knowledge sharing	The post shares general health or medical knowledge
Description of Information needs	Providing disease information	The post offers information or details about a particular disease
	Providing knowledge	The post provides general health or medical knowledge
	Citing information	The post quotes information from other sources
	Asking clear questions	The post articulates questions or concerns clearly
	Expressing emotions	The post expresses emotions or sentiments related to health issues

Then, the feedback types of the 9,951 replies were summarized using the similar steps, and combined with the posting content and reply content to determine whether each post received effective feedback, categorized as satisfied, partially satisfied or not satisfied. Some examples about the criteria for assessment are illustrated in the Table 3. In addition, responses are manually categorized into four types: answering, discussion, inquiring, and emotional supporting, depending on the content of the response.

**Table 3:** Examples about the criteria for effective feedback judgement.

Posting content	Information needs	Reply content	Effective judgement
I have optic nerve atrophy, what should I do?	Seeking treatment	You can drink a brain-enhancing and vision improving soup	Satisfied, effective feedback
Can someone recommend a experienced traditional Chinese medicine doctor for me?	Seeking famous doctors	Each traditional Chinese medicine doctor has their own expertise, so it may be difficult to make recommendations based on your description.	Not satisfied, ineffective feedback

### Influencing factor analysis

Logistic regression is a widely-used method for analyzing the influencing factors of categorical variables, particularly when the dependent variable is binary. In this study, binary logistic regression analyses were employed to assess the relationship between the characteristics of the post and whether the post received effective feedback. Specifically, most independent variables were derived from the coding process above. For the dependent variable, effective feedback was defined as either fully satisfying or partially satisfying the information needs expressed in the post, which is represented as "1". Conversely, if the post’s information needs were not satisfied, it was coded as "0". Detailed information on the independent and dependent variables will be shown in the result part, and for a better explanation of the regression model, the variables’ details will be explained just before the regression model results. As a final step, we conducted robustness tests by replacing variable and selecting subsample. Statistical analyses were conducted using SPSS version 22.0 software.

## Results

**Fig 2:**
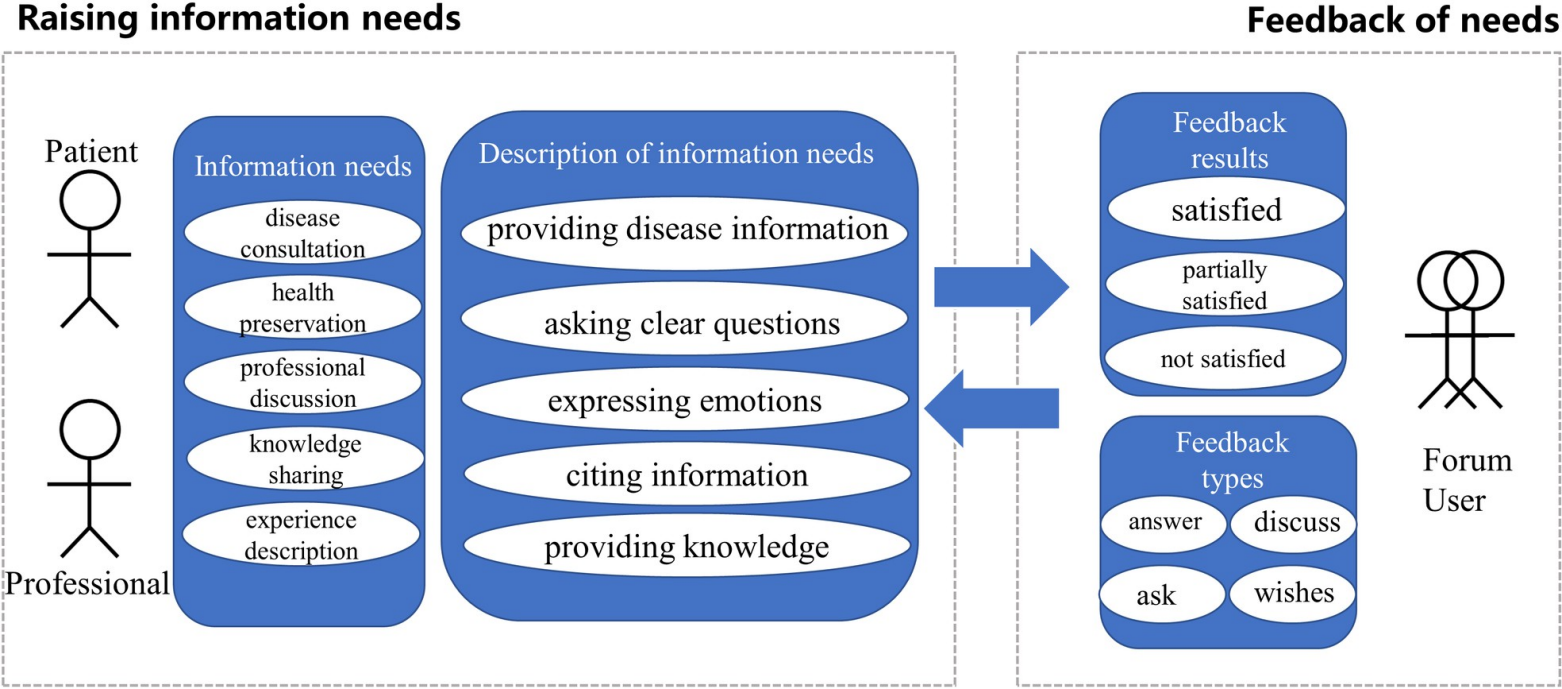
Information needs and feedback model of online TCM health community users.

Based on the coding results of the content analysis, this study constructed an information needs and feedback model for users of the online TCM community, as shown in Fig 2. The diagram is mainly divided into two sections: raising information needs and feedback on needs. The section on raising information needs includes the demanders represented by patients and professionals, the information needs consisting of 5 categories (disease consultation, health preservation, professional discussion, knowledge sharing and experience description), and the description of information needs consisting of 5 categories (providing disease information, asking clear questions, expressing emotions, citing information and providing knowledge). The feedback section is composed of two parts: feedback results and feedback types.

### Information needs

#### Demander

The demanders in the online TCM community can be roughly divided into 2 types, patients and professionals, as shown in Table 4. Among the 1,000 samples, 980 posts reflected the identity characteristics of the posters, and the content analysis results revealed that the online TCM community’s demanders included the following:

**Table 4:** Results of demander coding.

Primary category	Secondary category	Frequency
Patient-related demanders	Patients themselves	707
	Patient’s family and friends	182
	Other related people	4
Professional-related demanders	Medical staff	67
	Medical students	19
	TCM enthusiasts	13

(1) **Patient-related demanders,** including patients themselves, patients’ relatives and friends, as well as other related people, accounted for 90.9% of the total. Among them, patients accounted for 72.1% of all posts, while 18.6% of the posts were posted by patients’ relatives or friends seeking consultations. For example, the poster with ID 429115 saw the corpse case in the social news and was moved to post a consultation on how to treat cerebral palsy. *"The Nanjing girl’s corpse case was solved because the girl was suffering from cerebral palsy and multiple treatments were ineffective*, *so her father and grandfather*, *two elders*, *pushed her into the river*. *The reason for this is the fact that the girl’s body was found in the river*. *This news is very heartbreaking*, *and I feel the same way because there are also patients with major diseases in the family*. *Can you tell me if cerebral palsy is treatable*? *How can I treat it with Chinese medicine*?*"*

(2) **Professional-related demanders,** including doctors, nurses, medical students, and TCM enthusiasts, accounted for 9.6% in total. For example, the poster with ID 366993 wanted to communicate with other professionals through this platform. *"Hello everyone*, *I am a medical student studying TCM in Canada*. *Today*, *I met a patient with hypothyroidism*, *but since I haven’t graduated yet*, *I recommended a TCM doctor to her*. *However*, *I’m curious to know if I’m right about this case*…*"*

#### Types of information needs

According to the different content, the information needs of online TCM community users can be divided into 5 types: disease consultation, health preservation, professional discussion, knowledge sharing and experience description. The coding results are shown in Table 5.

**Table 5:** Coding results of the type of information needs.

Primary category	Secondary category	Frequency	Primary category	Secondary category	Frequency
Disease Consultation	Seeking treatment	393	Health preservation	Herbal cuisine	66
	Etiological diagnosis	250		Diet therapy	5
	Known treatment evaluation	144		Exercise	2
	Consultation	45	Professional discussion	/	62
	Seeking famous doctors	41	Knowledge sharing	/	17
	Other related consultations	6	Experience description	/	5

#### (1) Disease consultation

Disease consultation is the most common health information need of patient-related demanders. Specifically, patients seeking disease treatment methods account for the largest proportion, at 39.3%. The second most common information need is asking for a diagnosis, with patients hoping that doctors can provide detailed diagnoses or cause explanations based on symptoms and conditions (25.0%). A total of 14.4% of patients seek advice on forums based on known diagnoses and treatment plans, such as whether the prescribed medication is appropriate and whether other doctors’ diagnoses are accurate. A total of 4.5% of patients wanted to know the basics about their own disease, such as whether it could be cured and whether surgery was necessary. Another 4.1% of patients hoped to obtain more information about famous offline doctors from online forums. In addition, there are other disease-related consultations, such as disease treatment costs, medical insurance reimbursement, and filling out medical consultation forms, which account for 0.6%.

#### (2) Health preservation

The concept of "treating the unwell before they are sick" in TCM has deeply penetrated people’s minds. The information needs of users of the online TCM community in terms of health preservation, disease prevention, and treatment account for 7.3% and can be specifically divided into 3 types: information needs related to medication, diet and exercise. The application of medication for nourishing the body and adjusting its functions is suitable for a wide range of diseases and people, making it the most popular information need among these 3 types. Diet and health refer to the use of food based on its taste and characteristics to specifically treat certain diseases, help the body adjust and provide nourishment. Due to the small side effects of diet therapy, it is often the subject of consultations by elderly and for young children. The information needs for exercise often come from middle-aged and young patients who want to lose weight or improve their sexual health.

#### (3) Professional discussion

Professional discussion is mainly a discussion of clinical case records, medical or pharmacological knowledge, and professional terminology by clinical staff, medical students, or TCM enthusiasts. Common expressions like, *"I have a patient*…*" "Let’s talk about our opinions*.*" "I am a beginner*.*" "Please guide me*, *teacher*.*"* For example, *"Both the Bu Zhong Yi Qi Tang and Sheng Xian Tang can raise the middle and benefit Qi and lift Qi that has fallen*. *What are the similarities and differences in their effects*? *How do we choose and apply them clinically*? *Please share your opinions*.*"*

#### (4) Knowledge sharing

The main purpose of such users’ posts is to share or provide information related to TCM, such as knowledge about disease prevention and control, remedies and treatment methods, and professional interpretation of diseases.

#### (5) Experience description

A total of 0.5% of users do not have a clear inclination to ask questions or seek help but rather see the online health community as a platform to record their own or their friends’ experiences and treatments to provide some references for other patients with related conditions. For example, a patient with enteritis recorded his daily diet and health status for 1 month: *"Enteritis for more than 10 years*, *aggravated in the last 2 years*. *After staying at home for more than a year*, *I decided to take more than 1*,*000 yuan and go to the city to find a job*. *Diet record 09-12-05*: *white rice*, *winter melon*, *ginger*, *health condition b; 09-12-06*: *white rice*, *bean curd*, *c*. *Note*: *health condition is temporarily divided into 5 levels*, *"a*, *b*, *c*, *d*, *and e*.*"*

#### Description of information needs

To effectively express their needs, users need to provide various types of information through different ways of description (Table 6). Generally, when describing their needs, users may provide information about their medical condition, such as knowledge or references to traditional Chinese medicine classics, news, etc. Most users choose to ask specific questions when seeking consultation, while some users may express their subjective emotions more explicitly when describing their needs.

**Table 6:** Coding results of the description of information needs.

Primary category	Secondary category	Frequency	Primary category	Secondary category	Frequency
Providing disease information	Disease status	828	Asking clear questions	/	691
	Past medical history	552			
	Personal basic information	424	Expressing emotions	/	31
	TCM four diagnostic methods	100	Citing information	Citing TCM classics	18
				Citing news	4
	Tongue diagram	77	Providing knowledge	/	14
	Other	42			

#### (1) Providing disease information

Most of the users provided more information about their conditions before asking questions, including their basic personal information, symptoms of the disease, and histories of previous illnesses, medical consultations, treatments, and medications. In addition, the analysis revealed that 10.0% of the users provided information on the four diagnostic methods of TCM, such as cold and heat symptoms, tongue coating, pulse condition, and urination. For example, the user with ID 430903 attached a picture of the tongue coating after describing his symptoms. *"Male*, *29*, *with short yellow urine*, *occasional tinnitus*, *with mild bad breath*, *fat tongue with teeth marks*, *bags under the eyes*, *cold and drowsy feet in the afternoon*, *fatigue*, *sweats very easily (drinking hot and cold water and light exertion also cause sweating)*. *The following is the tongue coating photo*. *Ask your masters what is this disease*? *How should I take care of it*?*"*

#### (2) Asking clear questions

The results showed that 69.1% of users clearly expressed their specific consulting purpose and inquiry content in the needs description in the form of questions, and the common expression forms were *"*… *what’s wrong*,*" "how to treat*,*" "does it work"*, *"can it be”*, *etc*. The clear form of the question helps the replier understand the needs of the poster and provide a more targeted answer.

#### (3) Expressing emotions

Only 3.1% of users have obvious subjective emotions in their posts, often using exclamation marks or the words *"anxious"*, *"disappointed"*, *"pleading"*, etc. For example, the user with post ID 233774, who has not been cured after several treatments, could not help but complain while recording the progress of his condition: *"It’s a tragedy that a small inflammation has caused this*!*”*

#### (4) Citing Information

The content analysis results show that some users will quote the original sentences in TCM ancient books to illustrate the basis of their treatment or to draw discussion, mostly seen in the consultation questions of TCM enthusiasts or beginners, such as this one. *"A few days ago*, *I read the book Discernment of Internal and External Injuries*. *It was mentioned in the four times of medicine plus and minus method that if there are tingling pains in the abdomen or around the body*, *there is a lack of blood astringency*. *I suddenly remembered that I once had that problem*. *Is it also related to blood*?*"* There are also users who quote news events to introduce their own consultation questions. For example, the user with forum ID 193892 tried to find a famous doctor in the news through the platform, *"I am a Heilongjiang native*, *and I saw a story on TV about an old Chinese doctor in Heilongjiang who cured a little girl in Sichuan*, *and it only took half a month to cure the child’s strange disease*. *Now I want to look for that kind-hearted Chinese doctor to cure my loved ones*, *but I don’t know how to find him"*.

#### (5) Providing knowledge

This is commonly seen in knowledge-sharing types of posts, where users providing knowledge information tend to be more objective and use fewer emotional words, using more medical-related professional terminology, especially TCM terminology and theories. For example, a user with ID 362492 explained jaundice this way: *"Jaundice is mostly caused by damp heat in the liver and gallbladder*, *stagnating inside*, *unable to pass through*, *and blocking blood flow*, *causing yellowing of the whole body*. *Dampness and evil blockages inside the body*, *lead to weakened spleen yang and bile obstruction caused by dampness*, *which overflows onto the skin and turns yellow*. *Now*, *this will be a strange recipe for the world*: *Willow branch leaves in 20 grams of boiled water can resolve the condition*.*"*

### Feedback on information needs

#### Feedback results

This study also analyzed the 9,951 replies of the1,000 posts and coded whether the needs of each post were met. The statistical results showed that 78.8% of health information needs were effectively fed back. In addition, 21.2% of the needs were not met, with the most common reason being the lack of reply to the post. Other reasons included incomplete information provided by the poster, difficult-to-answer questions, and replies that did not address the original question.

#### Feedback ways

Based on the information and consultation questions provided by the posters, the responders gave feedback in 4 main ways: answering, discussing, inquiring, and emotional supporting.

#### (1) Answering

Above 60% of the responders directly answered the questions of the demanders. Sometimes, the demander’s consultation question contained multiple needs, and the repliers may not have answered all of them but may have selectively answered some of them. For example, user ID 426432 described his or her medical history and current symptoms in a post and expressed needs for both etiology and treatment methods. Reply 1 addressed the poster’s etiology needs by giving his or her own opinion, while Reply 2 provided a specific treatment prescription.

*Posting: "… I was under the care of an old Chinese medicine doctor two years ago and took Guizhi Longgu Muli Tang as a basic prescription for nearly half a year, which led to abnormal heart function. After searching, I suspected that the "Guizhi" in the Chinese medicine might have caused the problem, and many materials also reflect that patients taking drugs containing Guizhi may have abnormal heartbeats. I would like to ask, Is it Guizhi or the prescription that caused the discomfort? How can it be treated? Thank you."*

*Reply 1: "If you have tooth marks on the tongue, the tongue is wet and greasy, there are often bruises on the edges of the tongue, and there are cracks in the tongue, it is not right to take Longmu in this situation!"*

*Reply 2: "Tonifying qi, nourishing blood, and strengthening essence,. I have drafted a prescription for your reference: 15 grams of ginseng, 60 grams of roasted astragalus, 20 grams of dried atractylodes, 10 grams of citrus peel, 10 grams of dried ginger, 6 grams of cinnamon bark, 30 grams of angelica, 20 grams of pitted red dates (ground), 20 grams of Poria cocos, 15 grams of Polygala root, 30 grams of prepared Rehmannia root, 20 grams of dogwood fruit, 3 slices of fresh ginger, and 3 jujubes (without the core), decocted in water and taken twice a day."*

#### (2) Discussion

About 25% of the replies did not directly answer the questions of the demanders but expressed their own opinion by agreeing or disagreeing with the views of other repliers and having discussions with them, which provided additional information to the demanders. For example, in response to Reply 1 of the above post, a user quoted the original sentence and expressed his opposing viewpoint, *“[Tooth-scarred tongue*, *wet and greasy tongue*, *often bruises on the edge of the tongue*, *cracks in the tongue*, *easy to have loose stools—If this is the condition and you are still eating Longmu*, *it is not right*!*] "*

#### (3) Inquiring

Close to 10% of replies requesting more information from posters are commonly found in the following two situations. First, some responders wanted to answer the posters’ questions, but the information currently provided by the posters was not enough to support the posters’ direct answers. For example, to address the following posters’ needs, the responder asked him to provide specific information such as pulse and other medications.

*Posting: "Patient is 52 years old, male, diagnosed with atrophic gastritis by Western medicine. Every time he eats a little too much rice, he gets painful distention of the ribs and belches, and his tongue is almost gone. Can I use Mu Xiang Shun Qi Wan with Yu Nei Decoction?"*

*Reply: "What about the pulse? What about other medications?"*

The second situation is that some patients or family members of patients with the same confusion as the poster are asking about the poster’s recent condition, existing remedies, or treatments to satisfy their own needs for disease consultation. For example, in response to post ID 4264322, a user quoted his prescription and further asked the poster about his current situation: *"I have the same symptoms*. *Did the owner take it*? *And*, *how does he feel*?*"*

#### (4) Emotional supporting

Only about 3% of the replies expressed emotional support, such as comfort, encouragement, and well wishes, to the posters. These replies were common when the posters themselves described their needs with some pessimistic emotional overtones. For example:

*Posting: "If I get well, this disease is gouty arthritis. Even if it is difficult to accept, I have to accept. I can only say that it is a hard life. I often explain that God has exchanged the order of birth, old age, sickness and death."*

*Reply: "God loves stupid children. Be optimistic. It will get better."*

### Factors influencing the effective feedback of information needs

For answering RQ3, a regression analysis model was built based on the labeling results in the previous two sections to explore the core influencing factors of whether the information needs can get effective feedback.

#### Definition and description of variables

The independent and dependent variables in the regression model are taken from the labeling results above.

1. Dependent variable
  *Effective feedback*. As explained earlier in the feedback results section, this study manually labeled all the replies to 1000 posts. The dependent variable *Effective feedback* = 1 if the labeler believes that the post received at least one effective feedback on the information need, and *Effective feedback* = 0 otherwise.
2. Independent variable
  We analyzed the description characteristics of information needs in posts (Table 6). In this section, these characteristics will be used as independent variables.
  *Providing enough information*. This is a continuous variable. As shown in Table 6, users may provide six different types of information in posts such as “Disease status”. The independent variable *Providing enough information* is the number of types of information provided by the user and takes values ranging from 0 to 6.
  *Providing knowledge*. If knowledge information is provided in the post, take 1, otherwise take 0.
  *Citing information*. If citation of classic medical books or news is provided in the post, take 1, otherwise take 0.
  *Explicit questions*. If the question in the post is clearly articulated, take 1, otherwise take 0.
  *Expressing emotions*. If the posts express emotions, take 1, otherwise take 0.
3. Description of variables
  The results of the descriptive statistical analysis of the variables in the regression analysis model are shown in Table 7.

**Table 7:** Description of variables.

	Mean	Std	Min	Max
*Effective feedback*	0.788	0.409	0	1
*Providing enough information*	2.023	1.299	0	6
*Providing knowledge*	0.014	0.118	0	1
*Citing information*	0.022	0.147	0	1
*Explicit questions*	0.691	0.462	0	1
*Expressing emotions*	0.031	0.173	0	1

#### Logistic regression

The comprehensive test results shows that the χ2 value of the model is 22.378 with a p value of 0.000. Table 8 shows the result of logistic regression analysis. The model, at a significance level of α = 0.05, screened out 1 variable that had a significant effect on whether the health information needs of online TCM community users were satisfied, namely, providing enough information. The variable providing enough information is a numerical independent variable with a coefficient of 0.261, indicating that more information provided can positively influence whether the posters’ needs will be satisfied.

**Table 8:** Results of logistic regression.

Variables	B	S.E.	Wald	df	Significance	Exp(B)
*Providing enough information*	0.261	0.068	14.839	1	0.000	1.299
*Providing knowledge*	-0.306	0.604	0.256	1	0.613	0.737
*Citing information*	1.201	0.751	2.558	1	0.110	3.324
*Explicit questions*	-0.037	0.191	0.037	1	0.847	0.964
*Expressing emotions*	0.016	0.468	0.001	1	0.973	1.016
*Intercept*	-0.047	1.234	0.001	1	0.970	0.954

#### Robustness tests

This study tests the robustness of the regression analysis model by two methods.

1. Replacing variable. As shown in Table 8, only *Providing enough information* among the independent variables has a significant effect on the dependent variable. The number of types of information provided is usually related to the length of text, so we reconstructed the regression equation using *Length of post* as a replacement variable for *Providing enough information*. As shown in Table 9, the results of the regression analysis after replacing the variable are similar to those in Table 8. Only *Length of post* positively affects whether effective feedback is received. This shows that providing more information in posts does help to get effective feedback.
2. Selecting subsamples. In order to assess the effect of changing samples on the results, 500 samples were randomly selected from the 1000 samples to be analyzed. As shown in Table 10, the independent variable *Providing enough information* remained significant and the sign of the coefficient did not change. This random sampling was carried out 10 times and the results all showed that the regression analysis was robust.

**Table 9:** Robustness test results (replacing variable).

Variables	B	S.E.	Wald	df	Significance	Exp(B)
*Length of post*	0.001	0.000	15.003	1	0.000	1.001
*Providing knowledge*	-0.958	0.586	2.675	1	0.102	0.384
*Citing information*	0.845	0.760	1.237	1	0.266	2.328
*Explicit questions*	-0.082	0.185	0.194	1	0.659	0.922
*Expressing emotions*	-0.043	0.468	0.008	1	0.927	0.958
*Intercept*	1.321	1.137	1.350	1	0.245	3.749

**Table 10:** Robustness test results (selecting a subsample).

Variables	B	S.E.	Wald	df	Significance	Exp(B)
*Providing enough information*	0.215	0.086	6.217	1	0.013	1.240
*Providing knowledge*	-0.464	0.762	0.370	1	0.543	0.629
*Citing information*	0.729	0.769	0.898	1	0.343	2.073
*Explicit questions*	-0.185	0.246	0.563	1	0.453	0.831
*Expressing emotions*	0.735	0.756	0.947	1	0.331	2.086
*Intercept*	0.292	1.533	0.036	1	0.849	1.339

## Discussion

This study summarized health information needs and feedback in the online TCM community through content analysis and explored the relationship between information needs characteristics and needs feedback based on logistic regression. The main findings are as follows.

1. As shown in the labeling result in Table 4, users in the online TCM community include not only patients and patient-related stakeholders but also medical professionals such as clinicians, nurses, medical students, and TCM enthusiasts, whose main needs in the community are to discuss clinical cases or share expertise. This is similar to the findings of Lu Y et al. [37], who identified stakeholders in the online English health community MedHelp and found that 92.56% of posts were made by patients or patients’ family and friends, while 7.43% were made by medical professionals. The results suggest that managers of online TCM communities should fully consider the needs of medical professionals as a user group and optimize the applicability of the community to the entire audience.
2. The classification results in Table 5 indicate that the health information needs of online TCM community users can be divided into 5 major categories and 12 subcategories, including disease consultation, health preservation, professional discussion, knowledge sharing and experience description. Similar to the previous studies [38], TCM community users also focus on topics such as treatment methods, diagnosis of diseases, and applicable drugs when asking questions. Also this study identified some unique needs of TCM community users that have been less frequently mentioned in previous studies. First, as shown in Table 5, health preservation is the second highest ranked information need in TCM community, which differs from the findings of existing studies. This suggests that users in this community have needs for prevention and treatment of diseases and health care, which is in line with the idea of "treating the disease before it happens" in TCM. Second, compared with other health communities that focus on Western medicine[39], users in this community are not concerned about biochemical examination because the knowledge system of TCM is different from that of Western medicine. Third, some users use the community platform to seek local doctors. Chinese medicine pays attention to looking, listening, asking and feeling the pulse(four diagnostic methods), and some of these diagnoses are difficult to perform online. However, users can use the online TCM community to find TCM practitioners who specialize in treating a particular disease and then undergo offline treatment.
3. Users of the online TCM community are more detailed in describing their needs and providing some information with TCM characteristics. First, by calculating the length of the post text, we found that the average length of posts in this community is 343 words, which is significantly more than 264 words in the existing related studies [40]. In addition, whether patients can clearly describe their conditions and ask clear questions is an important health literacy ability, and users of this community will not only provide basic personal information, disease status and past history but also provide the four diagnoses of TCM, tongue images, and even cite TCM classics (Table 6). This shows that most users have good health literacy. However, statistical analysis shows that only 69.1% of users post clear initial consultation questions. In this regard, online community managers should provide online consultation guidelines or develop specific consultation templates to remind users to clearly express their needs.
4. Through manual labeling of the replies, the study found that most community users received effective responses to their information needs, which mainly took the form of answers, discussions, inquiries, and emotional support. Previous research has summarized the types of feedback as answers, inquiries, and emotional support [19]. This study identified a new form of feedback, namely, discussion, which not only provided more comprehensive supplementary information to the inquirer but also promoted communication among community users, thus increasing community activity. In addition, in contrast to emotional support, which is more common in other communities, this community’s responders tended to use rational approaches such as answering and discussing to directly address users’ information needs.
5. Providing enough information can significantly and positively affect whether needs receive effective feedback. In our study, the variable "Providing enough information" is derived from the provision of disease information within the primary category, as indicated by the coding results in Table 6 describing information needs. This encompasses the extent to which patients provide content across disease status, past medical history, personal basic information, TCM four diagnostic methods, tongue diagrams, and other relevant pictures. This finding coincides with the results of Yuelin Li et al.’s study [41]. They analyzed patient‒doctor interactions on an online health platform and found that the more detailed users reported their health conditions and the more symptom words they included when describing their needs, the more positively they influenced the efficiency of the interaction. In online TCM communities, more detailed and specific descriptions of conditions make it much easier for doctors to conduct TCM syndrome differentiation. Moreover, users who offer diverse forms of information in their posts may enhance their chances of obtaining effective feedback. For instance, providing images such as tongue images may help doctors in diagnosis because it enables patients to visually describe their condition. Therefore, it is advisable for users to present information about their condition in as many different formats as possible when articulating their needs.

There are still some limitations in this study. First, although the Huaxia TCM forum has a large influence and sample representativeness, the research results still need to be tested and validated in other TCM forums. Second, the 1000 samples extracted in this study have a limited amount of data, which can be combined with manual rule extraction, supervised learning methods [42] and automated annotation based on existing annotations to expand the annotation sample size and improve model accuracy. Third, due to the consideration of privacy and other ethical issues, this study did not collect user’s identity information, and therefore did not study the relationship between the user’s identity characteristics and behavioral characteristics in depth. In the future, we will explore the possibility of obtaining more detailed data through in-depth cooperation with forums, in order to conduct in-depth research.
